# Dating apps and websites as tools to reach anonymous sexual contacts during an outbreak of hepatitis A among men who have sex with men, Berlin, 2017

**DOI:** 10.2807/1560-7917.ES.2019.24.21.1800460

**Published:** 2019-05-23

**Authors:** Claudia Ruscher, Dirk Werber, Janine Thoulass, Ruth Zimmermann, Matthias Eckardt, Christian Winter, Daniel Sagebiel

**Affiliations:** 1 State Office for Health and Social Affairs (LAGeSo), Berlin, Germany; 2 Postgraduate Training for Applied Epidemiology (PAE), Robert Koch Institute, Berlin, Germany; 3 European Programme for Intervention Epidemiology Training (EPIET), European Centre for Disease Prevention and Control (ECDC), Stockholm, Sweden; 4 Department for Infectious Disease Epidemiology, Robert Koch Institute, Berlin, Germany

**Keywords:** outbreak, hepatitis A, MSM, online, dating apps, implementation science, sexually transmitted infections, viral infections, men who have sex with men

## Abstract

**Background:**

In an outbreak of hepatitis A among men who have sex with men (MSM) in Berlin (2016 and 2017), patients frequently reported anonymous sex and use of dating applications to meet sexual contacts, hampering tracing and vaccination of contacts.

**Aim:**

Our objective was to evaluate dating apps and websites as a means of spreading prevention messages among MSM during the ongoing outbreak.

**Methods:**

Advertisements in different formats were placed on three MSM dating apps and eight websites for anonymous dating during three weeks in March and April 2017. We calculated frequency of ads shown and click-through rates (CTR) and investigated the independent effect of format and platform on the number of clicks using a negative binomial regression model. We evaluated the campaign’s impact using a survey among visitors of a large gay-lesbian street-festival in Berlin.

**Results:**

Overall, 1,920,180 ads were shown and clicked on 8,831 times (CTR = 0.46%). The multivariable model showed significantly more clicks on one dating app (incidence rate ratio (IRR) = 9.5; 95% confidence interval (CI): 7.7–12.2) than on websites and on full-screen ads (IRR = 3.1; 95% CI: 2.5–3.8) than on banner ads. Of 266 MSM who participated in the survey, 190 (71%) knew about the outbreak and 39 (15%) declared to have been vaccinated recently because of the campaign.

**Conclusions:**

Dating apps provided a means to rapidly reach and influence a substantial number of MSM in Berlin and should complement case-based contact tracing among MSM in outbreak settings. Clicking on ads depended on platform and format used.

## Introduction

Hepatitis A is an acute infection of the liver, usually with a self-limiting course. Hepatitis A virus (HAV) is predominantly transmitted from person to person via the fecal–oral route, or through ingestion of contaminated food or water [1]. Sexually transmitted hepatitis A outbreaks among men who have sex with men (MSM) have been frequently described [2-5]. The disease is vaccine-preventable and the vaccine is highly effective [6].

In Germany, hepatitis A vaccination recommendations by the Standing Committee on Vaccination include people whose sexual behaviour increases the likelihood of exposure to HAV (including MSM) and this is therefore covered by health insurance [7]. Similar to other western European countries, Germany is a low-endemicity area and annual hepatitis A incidence was 0.9 cases per 100,000 residents in 2016 [8]. In Berlin, 35–88 cases were reported annually between 2010 and 2016 (incidence: 0.1–0.4 cases/100,000 residents) with a balanced sex ratio. The main preventive measure in hepatitis A outbreak control is tracing of household contacts and other close contacts and their subsequent vaccination with a two-dose regimen of inactivated hepatitis A virus within 2 weeks [9].

After the EuroPride festival in Amsterdam in 2016, several European countries were affected by a large hepatitis A cluster, with circulation of three distinct strains of HAV genotype Ia (VRD_521_2016, RIVM-HAV16–090 and V16–25801) that involved more than 4,101 cases in 22 countries in the European Union (EU) until May 2018 [10]. Berlin was affected by this outbreak from mid-November 2016 to the end of 2017, peaking in early 2017 [11]. The outbreak in Berlin comprised 190 cases, 162 of them male. Enhanced surveillance, including information on sexual contacts, revealed that 78% of them were MSM. In detailed case interviews with a subset of cases, the interviewees often reported having multiple sex partners while they were infective, often anonymously, and facilitated by the use of geosocial networking smartphone applications (dating apps).

Tracing of sexual contacts can be challenging when there are multiple and or anonymous sexual partners. This is of particular relevance for local public health authorities, where staff, money and time resources are often limited. There is evidence that MSM seeking anonymous sexual contacts predominantly use Internet-based communication technologies which may therefore serve as appropriate tools to reach that population [12]. A recent study showed that MSM websites and smartphone applications are popular platforms to find sexual partners; in particular, Grindr, Scruff and PlanetRomeo are the three most popular apps across Europe [13]. The State Office for Health and Social Affairs (SOHSA) in Berlin launched a broad information campaign from 10 March until 31 July 2017 to interrupt transmission among MSM. This included the distribution of posters and postcards containing information about the outbreak and the importance of vaccination. Campaign material was distributed in gay clubs, darkrooms, saunas and other sex-on-premises venues in Berlin. Furthermore, we launched an advertising campaign on dating apps and MSM websites to inform MSM in Berlin, including those seeking anonymous sexual contacts, about the ongoing outbreak and personal prevention measures.

With the widespread availability of smartphones and Internet access, the use of mobile-accessible apps has increased dramatically over the last decade, and geosocial networking apps for MSM facilitating anonymous sexual activities are known as potential drivers of recent outbreaks of syphilis, shigellosis and hepatitis A in Europe [14-16]. However, there is limited evidence on how to use those tools effectively as an intervention during infectious disease outbreaks among MSM. We evaluated the use of dating apps and websites as a means of spreading prevention messages among MSM during an ongoing outbreak of Hepatitis A in Berlin. In addition, we tried to assess the overall impact of the campaign with a survey during a popular gay-lesbian festival in Berlin.

## Methods

**Figure 1:**
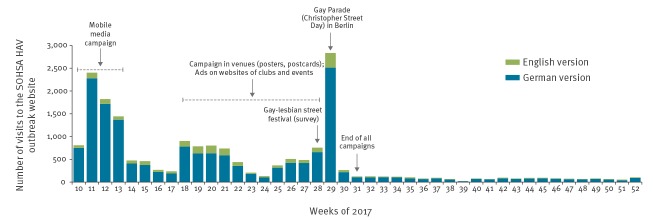
Page visits to the English and German version of the SOHSA outbreak website per week, in relation to campaigns launched during a hepatitis A outbreak among men who have sex with men, Berlin, 2017 (n = 17,163)

HAV: hepatitis A virus; SOHSA: State Office for Health and Social Affairs.

Our campaign consisted of three parts that took place on different and partly overlapping dates (as shown in the timeline in Figure 1): (i) a campaign launched on MSM dating apps and websites, (ii) advertising on websites of gay events and clubs and (iii) a poster and postcard-based campaign in clubs and sex-on-premises venues and during a gay-lesbian street festival. For the campaign on dating apps and websites, we calculated the frequency of ads shown and click-through rates (CTR) and investigated the independent effect of format and platform on number of clicks using a negative binomial regression model. We evaluated the campaigns´ success by their reach (i.e. number of impressions, number of clicks on an ad, number of page visits on the SOHSA outbreak website) and impact (knowledge of the outbreak in the MSM community and decline in the number of cases) using a survey at a gay-lesbian street festival and comparing the outbreak course with other countries.

### Campaign on dating apps and websites 

Three GPS-based dating apps used by MSM (Grindr, PlanetRomeo, Scruff), only accessible by mobile devices (i.e. smartphones, tablets), and a bundle of eight websites accessible by mobile devices and desktops were included in the campaign over a period of 3 weeks. Ads on Grindr, PlanetRomeo and the website bundle were displayed with slightly differing running times between 10 March and 1 April 2017 (week 10–13, the study period), targeted at users whose GPS was located within the geofence of Berlin at the time the ad was shown. Geofencing of ads shown on the websites was based on browser submission of location (city) when a website was accessed.

All ads were linked to a website of the SOHSA in Berlin containing information in German and English about the ongoing hepatitis A outbreak, HAV transmission routes, risk factors specific to MSM and recommendations for insurance-covered HAV vaccinations for MSM. The number of times an ad was displayed on an app or website while visited by a user was expressed as ‘ad impressions’. Tracking and counting of daily click numbers during the campaign was done by providers of the apps and websites using tracking pixels of the ads.

**Figure 2:**
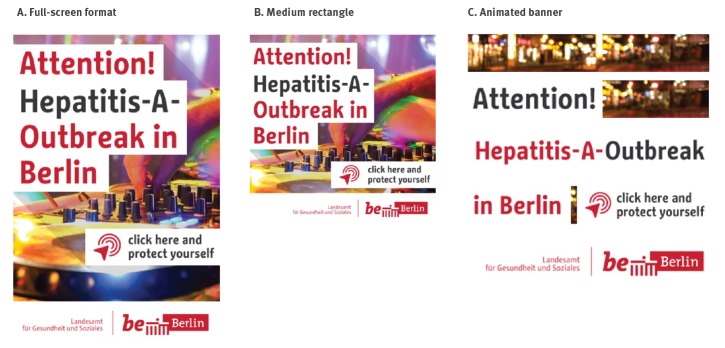
Ad formats used in a campaign on dating apps and websites about hepatitis A vaccination, Berlin, 2017

Ads were designed as GIF files in three different formats: a narrow banner (320 × 50 px for smartphones; 728 × 90 px for tablets and desktops), a medium-sized rectangle (300 × 250 px for smartphones; 400 × 400 px for tablets and desktop) and a full-screen format for smartphone use (320 × 480 px). All of them contained a flashing icon combined with the text line “*Click here and protect yourself!*” alternating in German and English (see Figure 2).

On Grindr, 200,000 banner impressions and 66,666 interstitial full-screen impressions were purchased. Interstitial full-screen ads covered the interface of their host app and were typically displayed at natural transition points in the flow of an app (i.e. between activities). The user had the choice to either tap on the full-screen ad and continue to its destination or close it and return to the app.

Over PlanetRomeo and the website package, 500,000 impressions of different partial and full-screen ads were distributed. The bundle of websites included in the campaign consisted of gay online magazines as well as MSM social networking sites and dating websites: www.blu.fm/, www.dbna.de/, www.gay.de/, www.planetromeo.com/de, www.queer.de/, www.queerpride.de/, www.siegessaeule.de/ and https://slamr.de/. 

Scruff uses a free ad platform (Benevolads), available for non-profit and public health causes. Free of charge, geo-targeted ads are created with a self-service tool that requires a Google account. Ads are designed by uploading a picture and inserting a text line. Using the account allows tracking of exact reach and engagement statistics (number of impressions and clicks) which are updated on a daily basis during the campaign. Start and end dates of the Scruff campaign were not adjustable but automatically set to 100 days; number of impressions displayed during the campaign was not adjustable either. To allow comparison between different digital media platforms, we included in the analysis only those statistics from Scruff that were obtained during the study period (10 March to 1 April). 

Results and statistics were recorded in spreadsheets containing the daily number of impressions, clicks and daily click-through rates (CTR; percentage of clicks among the total number of impressions), stratified by ad size and platform (each app and undifferentiated for the bundle of websites). Data were obtained from each of the apps and from a marketing agency for the websites.

### Multivariable analysis of the campaign on dating apps and websites 

Results from different sources were collated into one dataset containing number of impressions, number of clicks, CTR, ad size and platform specification by day of week. We considered the campaign on dating apps and websites as a cohort study of impressions where clicking on the ad was the outcome. Because of overdispersion of the count data (number of clicks), a negative binomial regression model (yielding incidence rate ratios (IRR)) was used to investigate the independent effect of ad format (banner, rectangle, full-screen) and mobile media placement (different apps vs website bundle) on click numbers (outcome variable). Multivariable analysis was restricted to data obtained during the study period, when the campaign involved all media platforms. Model selection was performed by using the Akaike information criterion (AIC) within a stepwise regression procedure. Statistical significance of the coefficients was determined using a z-score statistic. The model was adjusted for number of impressions and for weekdays to control for potential variability in user behaviour causing different numbers of impressions and clicks. Statistical analysis was carried out using Stata v.13.0 (StataCorp, College Station, Texas, United States).

### Additional free-of-charge advertising on websites of gay events and clubs

From 28 April 2017 onwards, organisers of the Christopher Street Day (a well-known LGBTQIA event that takes place every year at the end of July in Berlin) and owners of sex clubs and gay saunas in Berlin were asked to place one of the ads used in the digital campaign on their website and link to the SOHSA’s HAV outbreak website to inform their clients and visitors about the ongoing outbreak and preventive measures. None of those collaborations involved any payments or fees.

### Campaign in clubs and sex-on-premises venues and during a gay-lesbian street festival

Overall, 13,500 postcards and 250 posters, with the same design as the one used in the web campaigns, were distributed in gay clubs, darkrooms, gay saunas, pharmacies and shops between 5 May and 16 July 2017. They contained information in German and English about the outbreak and about vaccination as the most important prevention measure, as well as a shortlink to SOHSA’s HAV outbreak website. In addition, posters were tagged with near-field communication (NFC) chips, enabling mobile communication devices to connect automatically to SOHSA’s HAV outbreak website when within close proximity to the chip.

### Evaluation of the campaigns’ reach and impact

We evaluated the campaign’s success by its reach and impact. Outcome variables that derived directly from the campaign (i.e. number of impressions, number of clicks on an ad) were parameters to describe the reach of the campaign, defined as the number of people noticing and reacting to it. Daily numbers of page impressions and visits to the SOHSA HAV outbreak website were tracked between 10 March and 30 November 2017. Comprehensive website visitor tracking and traceback of the sources of website traffic was not possible. We could therefore not assign page visits to the SOHSA outbreak website to distinct sources (e.g. a specific website or dating app). Impact was measured by the indirect consequences of the campaign including knowledge of the outbreak in the MSM community and decline in the number of cases. We determined the impact of the campaign with two following approaches, a survey at a gay-lesbian street festival and comparison of the outbreak course with other countries. 

The overall cost invested for both the digital and on-site campaigning was used to calculate the amount spent per impression (digital and print material).

#### Survey at a gay-lesbian street festival

We conducted a convenience-sampled face-to-face survey among male visitors of the festival during a large annual gay-lesbian street festival on 15 and 16 July 2017.

The objective of the survey was to estimate the proportion of MSM who knew about the ongoing HAV outbreak, assess their source of information and estimate the proportion who got vaccinated as a consequence of the campaign. Regular use of dating apps, MSM status and nationality were also covered. Participants were given the choice of answering 10 short questions by themselves on a tablet computer or via the interviewer. Descriptive analysis focused on differences in demographic characteristics and source of outbreak information. We conducted logistic regression analysis to investigate the independent effect of different outbreak information sources on whether participants reported to have been vaccinated within the last 6 months because of the campaign. Statistical significance was determined at a significance level of p < 0.05. To estimate the proportion of MSM who knew about the outbreak, we calculated a sample size (n) of 185 using the equation n = (Z^2^ P(1-P))/d^2^, with a confidence level of 95% (Z = 1.96), a precision of d = 5% and an expected proportion of 14%. The expected proportion was based on the number of page visits to the SOHSA HAV outbreak website obtained until 6 July 2017 (n = 14,044) and an estimated size of the Berlin MSM population of 115,000 [17]. Anticipating a response rate of 60%, 308 visitors were needed.

#### Comparison of the outbreak course in Berlin and other affected countries in Europe

Routine and enhanced surveillance of HAV in Berlin was performed based on notification data, using the case definition for surveillance purposes in Germany [18]. Local public health authorities were requested to systematically collect additional information from HAV cases on sexual contacts and sex in non-household venues during the assumed infectious period [11]. Outbreak cases were defined as: a person notified with hepatitis A since 14 November 2016 and self-identified MSM or a person with an HAV outbreak sequence variant, or a person epidemiologically linked to such cases. Case numbers were compared with numbers of cases with an HAV sequence that belonged to the ongoing outbreak in MSM in other affected European countries, relying on surveillance data provided by the European Centre for Disease Prevention and Control (ECDC) [10].

### Ethical statement 

The street survey was conducted within the framework of the German Infection Protection Act as part of an outbreak response and public health practice. Mandatory regulations were respected, and thus review by an ethics committee was not required. Dating apps are location-based services and use of GPS data happens within the framework of their terms and conditions that users agree to. 

## Results

### Campaign on dating apps and websites 

From 10 March to 1 April 2017, a total of 1,920,180 impressions and 8,831 clicks on apps and websites were counted, reflecting an overall CTR of 0.5%. An average of 384 clicks on ads per campaign day was achieved along the 23 days of the campaign (Table 1).

**Table 1:** Ad impressions, clicks and click-through-rates, by platform and ad format, hepatitis A awareness campaign on dating apps and websites, Berlin, 10 March–1 April 2017

Digital media placement	Ad format	Impressions	Clicks	CTR	Campaign days during study period	Mean number of clicks per campaign day
Grindr	Full-screen	71,463	4,290	6.00	22	280.1
	Banner	215,196	1,873	0.87		
	Total	286,659	6,163	2.15		
PlanetRomeo	Full-screen	805	28	3.48	8	23.8
	Banner	206,035	162	0.08		
	Total	206,840	190	0.09		
Scruff^a^	Banner	1,132,248	1,767	0.16	22	80.3
Websites^b^	Banner	79,779	196	0.25	18	39.5
	Rectangle	214,654	515	0.24		
	Total	294,433	711	0.24		

CTR: click-through rate.

^a^ Ads on Scruff were also shown after the study period and reached overall 5,915,275 impressions and 7,794 clicks (overall CTR = 0.13).

^b^ Websites included: www.blu.fm, www.dbna.de, www.gay.de, www.planetromeo.com/de, www.queer.de, www.queerpride.de, www.siegessaeule.de, https://slamr.de.

During the 23 days, apps contributed to 85% of impressions (n = 1,625,747 and yielded most of the clicks (8,120; 92%). Ads on Grindr yielded most of the clicks (n = 6,163; 70%). Ads on PlanetRomeo were displayed during eight single days, yielding 190 clicks (2%). On Scruff, more than 1 million impressions yielded 1,767 (20%) clicks during the study period. Evaluating the entire time that ads were placed on Scruff (it continued for altogether 100 days from 11 March to 18 June 2017), a total of 5,915,275 impressions and 7,794 clicks were achieved.

The website campaign with banner- and rectangle-sized ads ran over 18 days (10 to 27 March 2017) on eight websites, resulting in 711 clicks.

### Multivariable analysis

In the negative binomial regression model, both the platform and the ad format were independent predictors of the number of clicks. Using Grindr resulted in nine times more estimated clicks compared with websites (IRR = 9.5; 95% confidence interval (CI): 7.4–12.2; p < 0.001), whereas Scruff (IRR = 0.8; 95% CI: 0.5–1.3; p = 0.337) and PlanetRomeo (IRR = 0.4; 95% CI: 0.2–0.6; p < 0.001) resulted in significantly fewer clicks than websites (Table 2). Full-screen ads received three times more clicks (IRR = 3.1; 95% CI: 2.5–3.8; p < 0.001) than banner ads, whereas medium rectangles received twice the number of clicks (IRR = 2.0; 95% CI: 1.5–2.7; p < 0.001) compared with banner ads.

**Table 2:** Multivariable analysis investigating the effect of ad format and platform on number of clicks, hepatitis A awareness campaign on dating apps and websites, Berlin, 10 March–1 April 2017

Independent variables^a^	IRR	95% CI	p value
Platform (apps/websites)			
Grindr	9.5	7.4–12.2	< 0.001
PlanetRomeo	0.4	0.2–0.6	< 0.001
Scruff	0.8	0.5–1.3	0.337
Websites	Reference		
Ad format^b^			
Full-screen	3.1	2.5–3.8	< 0.001
Medium rectangle	2.0	1.5–2.7	< 0.001
Banner	Reference		
Intercept	5.3	4.4–6.5	< 0.001

CI: confidence interval; IRR: incidence rate ratio.

^a^ Adjusted for impressions and weekdays.

^b^ Fullscreen: 320 × 480 px; medium rectangle: 300 × 250 px for smartphones, 400 × 400 px for tablets and desktop; banner: 320 × 50 px for smartphones; 728 × 90 px for tablets and desktops.

### Additional advertising on websites of gay events and clubs and poster and postcard campaign in clubs and sex-on-premises venues and during a gay-lesbian street festival 

Effects of those parts of the campaign were measured by number of page visits on the SOHSA HAV outbreak website after the campaign on dating apps and websites (i.e. 28 April–31 July). Since those two campaign branches ran on partly overlapping timeframes, their individual effects on the SOHSA outbreak page visits cannot be clearly separated. From 28 April until 31 July, a total of 8,118 visits to the SOHSA’s HAV outbreak website were counted: 6,650 on the German version, and 1,468 on the English version. A large share (1,087 visits) was counted the day the Christopher Street Day parade took place in Berlin. More than five weeks before that day, we had placed an ad on the website for this event. 

The SOHSA’s HAV outbreak website received an average of 541 page visits per week between 28 April and 31 July 2017 (443 on the German version, 98 on the English version). Because the English version of the website was only accessible through the German version, the number of clicks counted on the English version was subtracted from the counts of the website in German language. From 1 August to 31 December 2017, once all campaign elements had ended, the visits dropped to an average per week of 58 on the German version and 14 on the English version (Figure 1).

### Cost of the campaign

The campaign on dating apps and websites cost EUR 2,722 in total, an average of EUR 0.14 per impression. In addition, approximately EUR 300 were spent on the design of ads and copyright of pictures used. An average of EUR 0.31 was spent per click. For the postcard and poster campaign in sex-on-premises venues and clubs, an average of EUR 0.12 per impression (single postcard/NFC-tagged poster) was spent, corresponding to a total cost was around EUR 2,900, including the cost for design and NFC tags.

### Evaluation

#### Course of the outbreak

**Figure 3:**
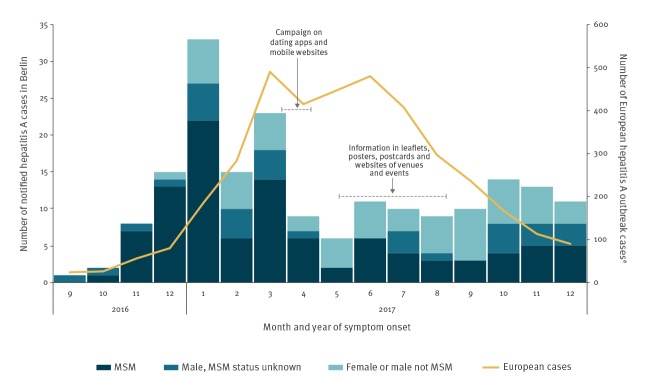
Confirmed hepatitis A outbreak cases^a^ by month of symptom onset, stratified by sexual orientation and sex, Berlin, Germany (n = 190), compared with cases in MSM in EU/EEA countries (excluding Germany), September 2016–December 2017

EEA: European Economic Area; EU: European Union; HAV: hepatitis A virus; MSM: men who have sex with men.

^a^ Confirmed outbreak cases in Berlin were cases according to the case definition (a person notified with hepatitis A since 14 November 2016 and self-identified MSM, or a person with an HAV outbreak sequence variant, or a person epidemiologically linked to such cases); confirmed cases in other EU/EEA countries were cases with an HAV sequence that belonged to the ongoing outbreak in MSM.

In Berlin, cases per month declined from an average of 18 outbreak cases during the peak months January to March 2017 to an average of six cases from April to December 2017. Case numbers in other affected European countries (21 countries, excluding Germany) peaked from March through June with an average of 458 cases per month and started to decline in July and August (average case number per month from July to December 2017: 219) (personal communication: Margot Einoder-Moreno and Ettore Severi, ECDC, May 2018) (Figure 3).

Where month of onset was unavailable, month of sampling or month of receipt by the reference laboratory was used. Countries included: Austria (until November 2017), Belgium, Croatia, the Czech Republic, Denmark, England, Estonia, Finland (until November 2017), France, Greece, Ireland, Italy, Latvia, Luxembourg (until November 2017), Malta, the Netherlands, Norway, Portugal, Slovenia, Spain and Sweden.

Source: Data kindly provided by Margot Einoder-Moreno and Ettore Severi from the ECDC, 24 May 2018.

### Survey

More than 350,000 people were estimated to have visited the gay-lesbian street festival. A total of 338 men were asked to participate in our survey about hepatitis A knowledge, of whom 292 agreed (response rate: 86%). A total of 266 (91%) self-reported as MSM. The median age of MSM was 39 years (range: 16–73 years; interquartile range 29–48 years) which was similar to the age distribution of cases in the hepatitis A outbreak in Berlin (data not shown). Overall 171 MSM (64%) reported to be regular users of dating apps (Table 3).

**Table 3:** Survey about hepatitis A knowledge among men who have sex with men at a gay-lesbian street festival, Berlin, 2017 (n = 266)

		n	%	Binomial exact 95% CI
Knowledge of the ongoing hepatitis A outbreak^a^		190	71.4	68.8–79.6
Source of knowledge	Print media	68	35.8	29.0–43.1
	Friends	43	22.6	16.9–29.3
	General practitioners	34	17.9	12.7–24.1
	Facebook	32	16.8	11.8–22.9
	Posters and postcards	26	13.7	9.1–19.4
	Other	23	12.1	7.8–17.6
	Ads on dating apps	21	11	6.9–16.4
	Ads on websites	10	5.3	2.6–9.5
	Internet search	4	2.1	0.6–5.3
Knowledge of insurance-covered HAV vaccinations for MSM^a^		195	73.3	67.6–78.5
Source of knowledge	Campaign (web-based and in venues)	20	10.3	6.4–15.4
	Friends	24	12.3	8.1–17.8
	Physicians	87	44.6	37.5–51.9
	Other	50	25.6	19.7–32.4
Got vaccinated because of campaign		39	14.7	10.6–19.5
Regular dating app use		171	64.3	58.2–70.0
Other information				
Resident in Berlin in the last 3 months		198	74.4	68.8–79.6
German		211	79.3	74.0–84.0
Foreign-born		50	18.8	14.3–24.0
Median age (range)		39 (16–73)		

CI: confidence interval; MSM: men who have sex with men.

^a^ Multiple answers were possible.

Seventy-one per cent (n = 190) of MSM knew about the ongoing outbreak. The main sources of information about the outbreak among MSM were print media (n = 68; 36%), friends (n = 43; 23%) and general practitioners (n = 34; 18%) (Table 3). Twenty-one MSM (11%) knew about the outbreak from ads in apps and 10 (5%) from ads on websites, whereas 26 (14%) knew about it from posters or postcards in venues. The sources of information were age-dependent. MSM aged 38 years or younger reported to have received the information about the outbreak more often from friends (20%; p = 0.001) or Facebook (15%; p = 0.023) than those older than 38 years (8% and 8%, respectively). Overall 14.7% of MSM (n = 39) stated they had been vaccinated because of the campaign. MSM who were informed by their physician about the outbreak were more likely to be vaccinated (OR = 3.2; 95% CI: 1.40–7.26; p = 0.006) than those informed by other sources.

## Discussion

We investigated the use of dating apps and websites to disseminate prevention messages to MSM during an outbreak of hepatitis A in Berlin focusing on the influence of ad format and media platform used. A total of 8,831 clicks received during a 3-week campaign on dating apps and websites, a decrease of case numbers and a broad knowledge among the Berlin MSM community about the outbreak suggest that the campaign had a wide reach and notable impact. Measured in clicks, the reach of our digital media campaign probably outweighed the reach of other channels such as the distribution of information material in sex-on-premises venues and traditional contact tracing by local health authorities. We found that both ad size and platform used influenced the number of clicks on an ad and the use of full-screen ads and the dating app Grindr were most successful in achieving clicks. Being the largest international all-male mobile dating app, Grindr has been involved in health prevention and promotion in the past and enables its users to include information on their HIV status and the last testing date in their profile. It is conceivable that its users are more sensitised regarding health-related issues and that outbreak-related ads benefit from a general credibility concerning prevention campaigns for sexually transmitted infections. Higher click rates for full-screen ads were expected as they interrupt the user’s current activity and require action, which can however be accompanied by unintended clicks. Using more versatile analytics programmes in future interventions could provide more information on conversion rates (percentage of visitors who took action on the target website such as viewing a specific page, downloading a particular file, duration of stay on the website) and thus help quantify the actual reach, although data protection issues need to be considered.

Evaluation of the campaign’s impact was based on different parameters, one of them being the overall course of the outbreak. The marked decline in HAV cases among MSM in Berlin in mid-April (following the mobile media campaign) cannot be explained by the overall decline in cases in the European outbreak, in which peak and decline occurred later [10].

More than 70% of MSM surveyed during the street festival knew about the ongoing outbreak, and their knowledge was from different sources including our campaign. Fifteen per cent of MSM in the survey reported that they had been vaccinated against hepatitis A within the past 6 months as a consequence of our information campaigns (digital and posters or postcards), although bias through selective participation in our survey must be considered. MSM who had been informed by their physician (general practitioner or treating physician specialised in HIV and infectious diseases treatment) about the outbreak reported more frequently that they had been vaccinated against hepatitis A. This highlights the crucial role of physicians in vaccination awareness among MSM, but possibly also reflects the fact that medical advice usually directly precedes a vaccination and is therefore more readily recalled. Compared with print media and information from friends and physicians, ads on dating apps and websites seem to have played a minor role among the participating MSM as sources of information (11% and 5%, respectively). However, our survey was conducted 4 months after the campaign on dating apps and websites during the Gay Pride week (i.e. the week between the gay-lesbian street festival and Christopher Street Day), which received considerable attention in print media, television and on Facebook. This may have led to an underestimation of the role of dating apps in the survey.

According to health insurance refund claims, uptake of the recommended monovalent HAV vaccination among men older than 18 years increased markedly in Berlin during the outbreak. The uptake increased from 670 HAV vaccinations in the first quarter of 2017 (median in 2012–2016: n = 356) to 965 in the second quarter 2017 (median in 2012–2016: n = 306) (personal communication: Thorsten Rieck, Robert Koch Institute, May 2018). Taking delays for incubation period into account, there is an apparent temporal relationship of both HAV vaccination uptake and case decline with our campaign. However, quantifying the effect of the campaign on dating apps and websites remains difficult, as it took place in the context of a wide range of activities and interventions throughout the first half of 2017 in Berlin (including information and surveys among physicians, information of patients and the broader public via newspapers and radio). Furthermore, MSM visiting Berlin as tourists and getting vaccinated because of the campaign after their return home would not have been captured in our data on vaccination uptake.

Despite a strong decrease in the weekly hepatitis A case numbers in Berlin, the outbreak continued at a lower level throughout 2017, which underlines that, in the context of an international outbreak, interventions cannot rely on local efforts alone. They require concerted international interventions and communication channels tailored to specific target groups. This is of even more concern given the highly interconnected sexual networks among MSM in Europe. A mathematical model of HAV transmission in an MSM population estimated that HAV epidemics can be sustained until at least 70% of the population are immune to HAV; this is a motivation to promote HAV vaccination in high-risk groups such as MSM [19]. According to notification data (data not shown), the vast majority of the Berlin HAV outbreak cases were infected in Berlin, suggesting that the threshold needed to lower the average number of onward infections per HAV-infected person below one was not reached at the time.

The free placement of ads on websites of local gay clubs, saunas and events yielded a large number of page visits on the SOHSA HAV outbreak website, especially in connection with big events in the MSM community such as Christopher Street Day. We therefore consider this as a useful communication tool for future large-scale international events like the gay pride festivals occurring throughout Europe. Close collaboration with venues and organisers of large events as well as local public health offices to provide broad and up-to-date information channels for visitors was an integral part of the campaigns and the outbreak interventions. Future interventions should also consider and evaluate other potential ways of smartphone app campaigning such as sending inbox and push messages or video ads.

### Limitations

Because a part of the purchased ad bundles were not customisable, not all combinations of ad format and media placement were used and different formats were not evenly distributed over the platforms. The number of impressions or clicks does not correspond to the actual number of people receiving or clicking on the respective ads. Multiple displays of impressions on the same device were limited to a maximum of three for certain platforms (i.e. Grindr and PlanetRomeo), but not for others. Likewise, multiple clicks caused by the same user cannot be excluded. In addition, users who did not click on the ad may still have benefited from seeing the key message and have pursued the issue in other ways. Furthermore, it remains unknown to which extend the clicks of an ad reflect preventive action by the user. The major limitation however, is the evaluation of the campaign’s impact. HAV vaccination uptake provides a suitable parameter to evaluate the campaign’s overall success. However, disentangling single effects of multiple simultaneous interventions that were launched as part of an immediate outbreak response was not possible; we therefore had to use other, less well suited, surrogate parameters.

## Conclusions

Our findings suggest that MSM dating apps and websites can be an effective tool to promote infection prevention campaigns in outbreaks, with the potential to reach also otherwise hard-to-reach MSM seeking anonymous sex. The wide reach of our digital campaign – embedded in other outbreak control activities – was indicated by a decline in cases as well as an increase in HAV vaccination uptake and knowledge about the outbreak in the MSM community. The number of clicks and thus the overall reach of our digital campaign strongly depended on platform and format, which should be considered when launching an app-based health promotion campaign. Intervention strategies among MSM should include a variety of communication pathways and involve physicians in order to maximise impact in the outbreak setting.
